# Preliminary Study of Reinforced Glulam Beams with a High-Performance Composite Made of Polyvinyl Alcohol, Carbon Fiber, and Nanomaterials

**DOI:** 10.3390/polym18091018

**Published:** 2026-04-23

**Authors:** Mario Núñez-Decap, Marcela Vidal-Vega, Camila Opazo-Carlsson, Boris Moya-Rojas, Cecilia Fuentealba-Becerra

**Affiliations:** 1 Laboratorio de Productos de Ingeniería a Base de Madera y Adhesivos, Departamento de Ingeniería Civil y Ambiental, Universidad del Bío-Bío, Avenida Collao 1202, Concepción 4051381, Chile; mvidalve@ubiobio.cl (M.V.-V.); copazo@ubiobio.cl (C.O.-C.); bmoya@ubiobio.cl (B.M.-R.); 2 Centro Nacional de Excelencia para la Industria de la Madera (CENAMAD)—ANID BASAL FB210015, Pontificia Universidad Católica de Chile, Santiago 7820436, Chile; c.fuentealba@udt.cl; 3 Unidad de Desarrollo Tecnológico, Universidad de Concepción, Avenida Cordillera N° 3624, Parque Industrial Coronel, Coronel 4191996, Chile

**Keywords:** reinforced glulam beam, composite reinforced wood, carbon fiber-reinforced polymer

## Abstract

Engineered wood products manufactured with the durability and density of a *Pinus radiata* D. Don species usually do not achieve the mechanical properties of a structural material for construction; hence, the reinforcement of this kind of product is recommended, but the use of commonly used hazardous adhesives is a problem. Therefore, the primary objective of this research was to investigate the enhancement of various properties of glulam beams made from radiata pine through the application of a high-performance reinforcing composite, based on carbon fiber, polyvinyl alcohol, and other nanomaterials, at a laboratory scale. For this purpose, thermal and mechanical tests were performed in different composite formulations to choose the best ones and to manufacture the glulam beams, in which bending properties were measured. Based on the results, the samples reinforced with graphene stood out, and the samples mixed with epoxy resin presented statistically the same values of flexural stiffness and strength as the control samples elaborated with commercial wood adhesives. It is also important to highlight the performance of the samples M7 (PVA (7.5%) + NL (0.01%) + GP (0.01%) + NSiO_2_ (0.01%)) and M8 (PVA (7.5%) + NL (0.01%) + GP (0.01%) + NTiO_2_ (0.01%)), which are not mixed with epoxy resin and showed statistically the same flexural performance as epoxy resin, in terms of maximum load and displacement. As a conclusion, it could be said that this new high-performance composite could be a comparable alternative to hazardous commercial adhesives, by obtaining lower values, but close to those of the control sample, which are the most used when reinforcing wood products with engineering fibers.

## 1. Introduction

In Chile, several forest species have been introduced, among which radiata pine and eucalyptus stand out, currently forming the basis of national forestry development. *Pinus radiata* D. DON accounts for 57.9% of plantations according to the Chilean Statistical Yearbook of Forestry [1]. However, this species has low mechanical properties and durability [2], which limits its use as a structural material in the construction industry. Therefore, engineered products made from this wood species, such as glulam beams, are being reinforced to improve performance and increase wood use in construction. Among the solutions proposed in the last five years, research on reinforcement with engineered fibers and nanocomposites stands out. These mainly contribute to high values of bending strength, load-bearing capacity, stiffness, and density [3]. In 2025, Wdowiak-Postulak et al. [4] studied the reinforcement in bending of sheared wooden beams using pre-stressed natural and artificial fibers, achieving an enhancement of load-bearing capacity and stiffness with pre-stressed basalt bars and with natural fibers compared to wooden beams without any reinforcement. In 2025, Alaşalvar et al. [5] conducted an experimental and numerical investigation of the flexural behavior of old wooden beams strengthened with carbon fiber-reinforced polymer (CFRP). The results highlight the effectiveness of strengthening and preserving historic wooden structures. In 2025, Debnath et al. [6] studied the improvement of a timber beam’s performance through the reinforcement with carbon and glass fibers to enhance strength and water resistance, obtaining good results in tensile and flexural strength, impact resistance, and water absorption. In 2025, Bakalarz & Kossakowski [7] studied the improvement of the bending stiffness of timber beams using ultra-high-modulus CFRP sheets through experimental and numerical tests, thereby enhancing flexural stiffness and ductility. In 2024, Bakalarz [8] studied the mechanical properties of full-scale wooden beams strengthened with CFRP sheets to extend the life of existing wooden buildings and structures, obtaining improvements in load capacity, stiffness, and ductility. In 2023, the authors Ozdemir et al. [9] investigated the flexural behavior of laminated wood beams strengthened with novel hybrid composite systems made of carbon fiber-reinforced polymer (CFRP) and wire rope, which increased the maximum load and displacement compared to a control sample without reinforcement. The CFRP also prevented crack development. In 2023, Kılınçarslan & Türker [10] studied the strengthening of solid wooden beams with fiber-reinforced polymers to repair and strengthen elements damaged by fatigue and biological attack over time, rather than replacing them, achieving better results than unreinforced reference samples. In 2022, the authors Dániel et al. [11] studied the bearing capacity of reinforced glulam beams with CFRP plates, comparing an experimental design with numerical modeling. In 2021, the authors Ísleyen et al. [12] studied the behavior of glulam beams strengthened with CFRP to increase bearing capacity and improve overall load–displacement behavior. In 2021, Işleyen and Kesik [13] conducted an experimental and numerical analysis of the compression and bending strength of old wood reinforced with CFRP strips, obtaining a good correlation between the two.

This research focused on the manufacture of composite materials, which are formed by combining two or more materials that, when joined, create an element with distinct characteristics, properties, and mechanical behavior. To this end, materials such as carbon fiber, polyvinyl alcohol (PVA), and nanomaterials, including lignin, carbon nanotubes, graphene, silicate, and titanium dioxide, were utilized. The main characteristics of carbon fibers (CF) are light weight, stiffness, high tensile strength (TS), low thermal expansion, energy storage capacity, flexural stiffness and strength, heat resistance, and electrical conductivity [3,14]. Polyvinyl alcohol (PVA) is a highly polar, biodegradable, non-toxic, and water-soluble polymer commonly used as a polymer matrix for fabricating composite materials [15,16]. The materials were used at the nanoscale to increase surface area, thereby enabling better exploitation of each material’s properties [16,17]. Lignin is a biopolymer with properties that can promote high thermal stability, improved mechanical properties, resistance to decay and biological attacks, UV absorption, and high stiffness, among others [15,18]. On the other hand, carbon nanotubes (CNTs) are cylindrical carbon molecules that exhibit extraordinary strength, electrical, mechanical, and thermal properties [19]. Another carbon-based structure is graphene (GP), a two-dimensional crystal of carbon atoms that exhibits many properties, such as high electron mobility, enhanced thermal conductivity, exceptional mechanical strength, and optical and magnetic properties [20,21,22]. Other types of reinforcement to this composite are silicate and titanium dioxide nanoparticles. Sodium silicate solutions have excellent properties of adherence, film formation, and low cost [23]. Titanium dioxide possesses many properties, including structural, thermal, electronic, and optical [24].

The main goal of this study was to improve the mechanical properties of glulams elaborated with *Pinus radiata* D. DON wood species, through the reinforcement with a high-performance composite made of carbon fiber and nanomaterials (nanolignin (NL), carbon nanotubes (CNT), graphene (GP), and nano silicate dioxide (NSiO_2_) or nano titanium dioxide (NTiO_2_)) at laboratory scale. Research and results that are part of the execution of the FONDEF ID18I10038 project, entitled “Development of structural elements based on local wood, reinforced by a high-performance polymer composite, for construction applications in housing construction”, were funded by the National Commission for Scientific and Technological Research of Chile, between the years 2018 and 2022.

## 2. Materials and Methods

### 2.1. Materials

For the adhesive matrices, polyvinyl alcohol (PVA, CAS 9002-89-5), lignin (CAS 8068-05-1), sodium silicate (CAS 1344-09-8), titanium dioxide (CAS 13463-67-7), carbon nanotubes (CAS 308068-56-6), and graphene (CAS 7782-42-5) were acquired. Lignin, titanium, silicate, carbon nanotubes (CNTs), and graphene (GP) were purchased from Sigma-Aldrich (USA), and PVA was purchased from Merck. The epoxy resin (ER) used was Sikadur 330, a two-component system purchased from Sika. For the composite formulation, the carbon fiber was purchased from Aura Industrial (200 g/m^2^, bidirectional).

For the wood products manufacture, *Radiata pine* D. DON wood species was used, with an average density and moisture content of 460 kg/m^3^ and 10 ± 2%, respectively, without any defects, and it was provided by the Laboratory of Engineered Products based on Wood and Adhesives (PRODIMA-LAB), Universidad del Bío-Bío, Concepción, Chile.

### 2.2. Adhesive Formulation

#### 2.2.1. Preparation and Measurement of Nanoparticles

The preparation of nanolignin consisted of reducing the size of a 10.0% m/m lignin (L) solution in water using a dispersing device (homogenizer, IKA T25D) for 2 h at 10,000 rpm and 4 h at 20,000 rpm. The aim was to obtain an average size of 100–120 nm.

In the case of nano titanium, its preparation involved reducing the particle size of a 1.0% m/m titanium dioxide solution in water for 5 h at 10,000 rpm. The objective average size is 100–120 nm. And the nanosilicate preparation consisted of reducing the size of a 1.0% m/m sodium silicate solution in water for 2 h at 5000 rpm, followed by 1.5 h at 10.000 rpm. The aim was to obtain an average size of 70–80 nm.

The nanoparticles were measured using a Transmission Electron Microscope (TEM), Hitachi model HT7700.

#### 2.2.2. Preparation of Adhesive Bases

Eight wood adhesive bases (M1, M2, M3, M4, M5, M6, M7, and M8) were prepared using a heating plate at 120 °C, with agitation of 300 rpm, for 4 h. The adhesive matrix sample and five control adhesive blends (ER, P1, P2, P3, and P4), as specified in Table 1, were mixed with different proportions of epoxy resin, as specified in Table 2.

#### 2.2.3. Characterization of Adhesive Bases

The characterization and study of the adhesive bases were carried out by measuring viscosity, pH, and density. The viscosity was measured at 20 °C using a digital viscosimeter (Brookfield model DV2T) with spindle n°7, according to the ASTM D1084 standard [25]. The pH was measured at 18 °C using a pH meter (Hanna Instruments), according to the ASTM E70 standard [26]. Density was measured at 20 °C using a pycnometer in accordance with the UNE-EN 542 standard [27]. Measurements are taken in triplicate.

The thermal performance was evaluated using Thermo-gravimetric Analysis with Differential Scanning Calorimetry (TGA/DSC; PerkinElmer model STA 800). A quantity of 15–25 mg of each adhesive matrix was used. The samples were subjected to a temperature sweep from 30 °C to 1400 °C (due to the high degradation temperature of the carbon-based nanoparticles [28,29]) at a heating rate of 20 °C/min under a nitrogen atmosphere. The thermal degradation process of the samples was monitored. The results of the temperature and mass loss during the initial thermal degradation stage of the samples are presented. These parameters are useful to set the temperature range in DMA tests.

### 2.3. Characterization of the Composite

#### 2.3.1. Dynamical Thermo-Mechanical Analysis

**Figure 1:**
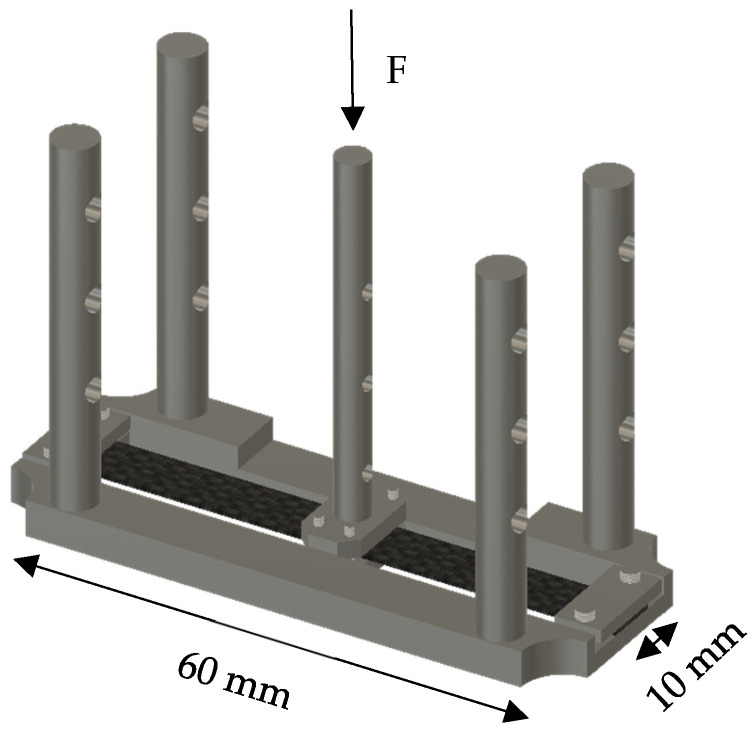
Dual cantilever device and specimen dimensions, for DMA test.

A dynamic thermo-mechanical analysis was performed on 4 samples, in duplicate, as described in Table 3, to evaluate the performance of the high-performance composite. This analysis was performed using a dual cantilever tool in a Dynamic Mechanical Analysis (DMA) instrument (Netzsch DMA 303 Eplexor). Samples of 10 mm width, 0.5 mm thickness, and 60 mm length were used (Figure 1). The samples were subjected to a temperature sweep from 30 °C to 400 °C at a heating rate of 30 °C/min and a frequency of 1 Hz. The storage modulus and tan δ were monitored along the test.

#### 2.3.2. Tensile Strength of Carbon Fiber Coated with Adhesive Bases

The tensile strength of carbon fiber coated with an adhesive matrix was tested to evaluate the effect of the matrix on the carbon fiber fabric.

**Figure 2:**
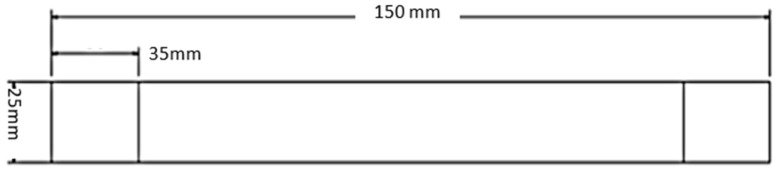
Specimen dimensions according to ISO 527-4 standard [30].

The test specimens were prepared in accordance with the UNE-EN-ISO 527-4 [30] standard for carbon fiber tensile testing, using type 1 B rectangular specimens (Figure 2). Pieces of wood 5 mm thick, 25 mm wide, and 35 mm long (two on the upper face and two on the lower face, four in total per test piece) were incorporated into the ends of these test pieces and adhered to the samples using epoxy adhesive (Sikadur 300) to facilitate their adjustment inside the clamps used in the tensile test.

Five specimens were tested for each composite to be evaluated, at a speed of 5 mm/min, using an Instron universal testing machine.

#### 2.3.3. Manufacture of Laminar Joints

The strength of radiata pine wood joints with composite adhesive bases was evaluated by manufacturing and testing them through a mechanical tensile shear test. In the first part of this study, laminar joints were fabricated to test the formulated adhesives and select the best ones for glulam beam fabrication. Hence, clear wood pieces without defects were chosen, with dimensions of 5 mm in height, 90 mm in width, and 400 mm in length. The two wood pieces were glued together, considering a grammage of 220 g/m^2^. Finally, the beams were cold-pressed at 12 bar for 24 h at 20 °C ± 5 °C. After gluing and pressing the wood pieces, the samples were stored at room temperature for 72 h. Then, they were cut, and 30 specimens per sample were obtained to evaluate the quality of the glue line across all formulations using a shear test under three different conditions.

#### 2.3.4. Shear Test of Laminar Joints

**Figure 3:**
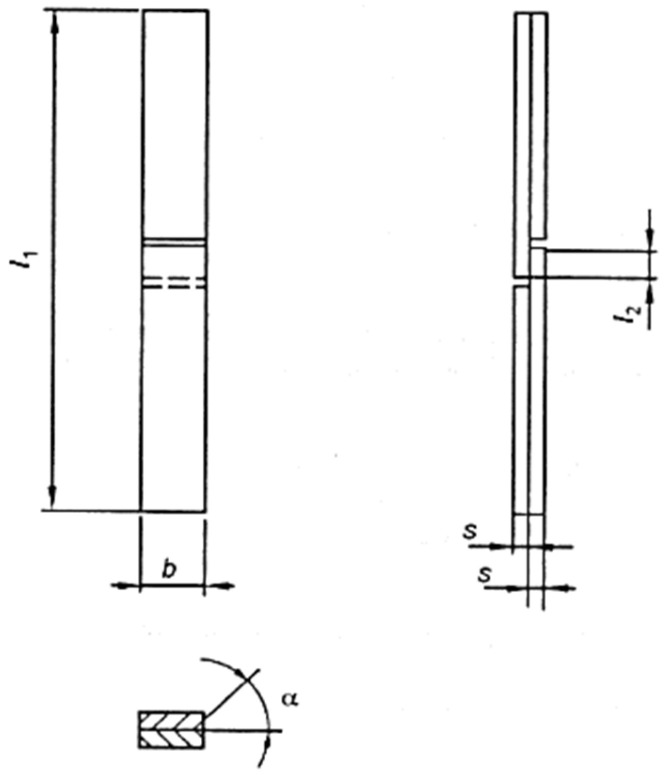
Specimen dimensions according to UNE-EN 205 standard.

The adhesive–wood bond was evaluated under the highest durability classification of the standard, class D4, which can be used in indoor conditions with frequent, prolonged exposure to running or condensed water and/or weather exposure, provided the surface is protected by a suitable surface coating. Then, the shear test was conducted according to the European standard UNE-EN 204/205 [31,32] specifications, using normal or dry conditioning cycles, boiling cycles, and a 4-day water-immersion cycle. A total of 30 specimens per sample were evaluated, 10 specimens for each condition, according to the dimensions shown in Figure 3. Where l1: 150 ± 5 mm: specimen length; l2: 10 ± 0.2 mm: length of the overlapping area; s: 5 ± 0.1 mm: specimen thickness; b: 20 ± 0.2 mm: specimen width. The requirements of the shear test according to the UNE-EN 204 standard are presented in Table 4.

The shear test was performed on an Instron 4468 universal testing machine at a loading speed of 50 mm/min until failure.

Based on the shear test results, two adhesive blends and two adhesive matrices will be selected for beam manufacturing.

### 2.4. Manufacture and Evaluation of Glued-Laminated Radiata Pine Beams at Laboratory Scale with Selected Adhesives

#### 2.4.1. Manufacture of Glued-Laminated Beams

**Figure 4:**
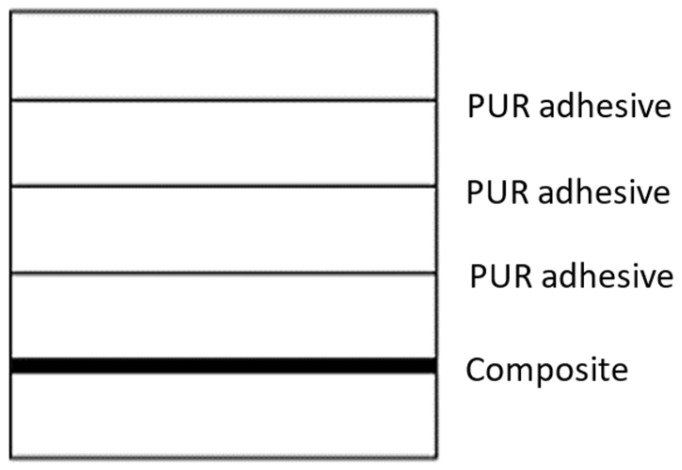
Configuration of reinforced beam assembly.

The glued-laminated beams were made from 5 layers of radiata pine wood, with dimensions of 550 mm in length, 30 mm in height, and 25 mm in width. A carbon fiber layer was incorporated between the 4th and 5th layers on the flexo-traction side in the reinforced beam samples (Figure 4).

Ten samples and six beams of each one will be manufactured, according to the composite identification described in Table 5.

The samples M5+ER, M6+ER, M7+ER, and M8+ER were elaborated considering a proportion of M/ER = 50%/50% m/m. The pressing parameters for all the samples were as follows: 14 g of adhesive quantity, 24 h of pressing time, and 9 bar of pressure.

#### 2.4.2. Physical Properties of Glulam Beams

The physical properties, density, and moisture content were measured for each sample according to the specifications in UNE-EN 323 [33] and UNE-EN 322 [34], respectively.

#### 2.4.3. Mechanical Properties of Glulam Beams

**Figure 5:**
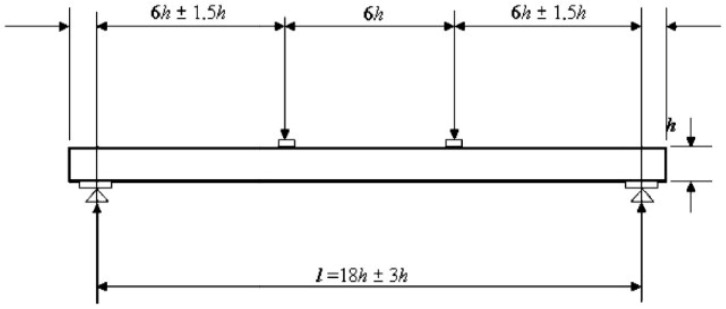
Specimen dimensions according to UNE-EN 408 standard [35].

The mechanical properties of glulam beams were analyzed using four-point bending tests in accordance with the UNE-EN 408 [35] standard. The four-point bending test was performed on an Instron 4468 universal testing machine at a preload of 0.1 kN and a loading speed of 4 mm/min until failure. The stiffness (MOE), flexural strength (MOR), maximum load, and displacement values were thus determined for each beam. Six specimens per sample were evaluated, according to the dimensions shown in Figure 5, where h = 30 mm.

#### 2.4.4. Statistical Analysis

The results were statistically analyzed using Statgraphics Centurion XV software via ANOVA (Version 15), with data distribution validated beforehand. Subsequently, a multivariate Fisher’s Least Significant Difference (LSD) test was applied to assess statistically significant differences in the physical and mechanical properties by comparing two means from two different groups by calculating the smallest significance as if a test had been run on those two means [36].

## 3. Results and Discussion

### 3.1. Measurement of Nanoparticles

**Figure 6:**
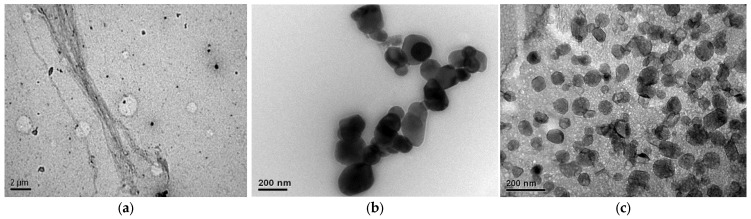
Micrograph of nanoparticles for measurement, (**a**) nanolignin (NL); (**b**) nano titanium dioxide (NTiO_2_); and (**c**) nano silicate (NSiO_2_).

From the preparation of nanoparticles and subsequent measurements, the intended average size of each was obtained, and micrographs of each sample are shown in Figure 6. In first place, the nanolignin presented a size of 118 nm, which is within the expected range (100–120 nm), in second place, the nano titanium dioxide average size is 105 nm, which is also within the predicted range (100–120 nm), and finally, the nano sodium silicate presented an average size of 77 nm, which is within the expected range too (70–80 nm).

### 3.2. Selection of Adhesive Bases

#### 3.2.1. Characterization of Adhesive Bases

The characterization results of the adhesive bases, in terms of viscosity, pH, density, and thermal characterization, are presented in Table 6.

From the physical and chemical characterization results for the adhesive bases, it can be observed that the samples’ viscosities ranged from 1020 cP (M3) to 2880 cP (M5). A tendency is observed among the samples P1, P2, P3, and P4, where viscosity increases with more nanoparticles; in P3 and P4, the sample with graphene (P4) had higher viscosity than the sample with carbon nanotubes (P3).

The pH of the samples ranged from 6.29 (P1) to 10.05 (M3). A tendency is observed between the samples M1, … M8, because the samples that have NSiO_2_ in their composition presented a higher pH value compared to their pairs that have NTiO_2_ in their composition.

The density of the samples ranged from 1.01 g/mL (M5, M7, and M8) to 1.06 g/mL (M1), with an average of around 1 g/mL.

From the thermal characterization results of the adhesive bases, it can be observed that the initial degradation temperature of the samples varied from 263.30 °C (M1) to 288.49 °C (P3), while the mass loss at the initial degradation of the samples varied from 49.03% (M1) to 84.97% (P4). A clear relationship between these parameters is evident: samples that required a lower initial degradation temperature showed lower mass loss, whereas samples that required a higher initial degradation temperature showed higher mass loss.

#### 3.2.2. Dynamical Thermo-Mechanical Analysis

**Figure 7:**
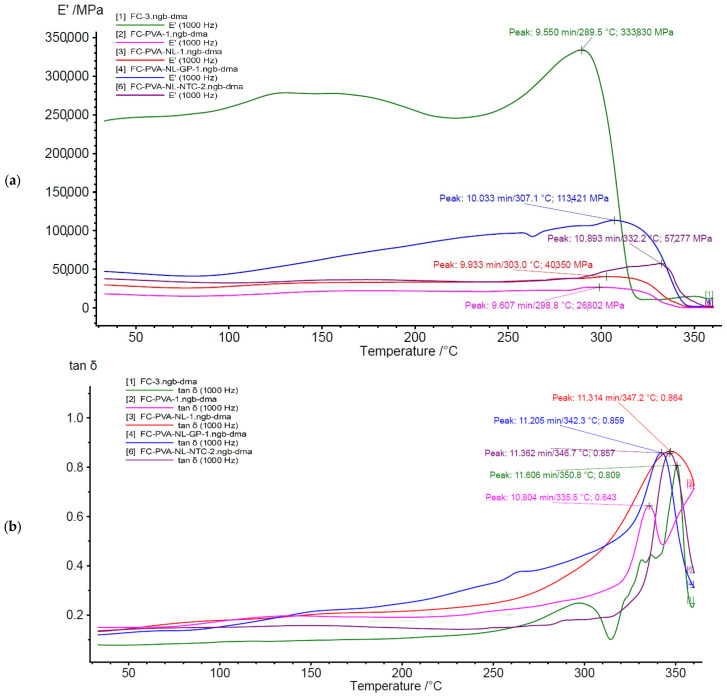
DMA (**a**) storage modulus and (**b**) tan δ curves.

The results of the dynamical thermo-mechanical analysis are presented in Table 7 and in Figure 7.

From the dynamic thermal analysis results of the composite samples, it can be observed that the storage modulus ranged from 26.80 GPa (CF-PVA) to 335.16 GPa (CF). In contrast, the temperature at which the peak of storage modulus was reached varied between 288.60 °C (CF) and 332.20 °C (CF-PVA-NL-CNT).

A significant difference can be observed in the results between the CF sample and the CF-coated samples, where the rigidity of the coated CF with PVA decreases, because of the loss of bond strength along with the temperature [37], incorporating elasticity into the composite, a characteristic of the thermoplastic adhesives [38], which was expected according to the storage modulus range in Table 8.

The temperature at which the rigidity decreases also increases when incorporating PVA, in most cases, which helps broaden the range of processing and applications [43]. This effect can also be observed in the investigation by Quan et al. [41] when PVA is incorporated into the composite. Additionally, it increases when the E′ decreases, representing the elastic behavior of the polymer [44]. On the other hand, it can be observed that the nanoparticles increase the rigidity of the composite CF-PVA, which is because the nanoparticles try to hold the composite in place, so a larger amount of energy is required to deform the samples [45], considering that the storage modulus is correlated with the stored energy [46]. The increase in stiffness, E′, was also observed in the investigation of Vidal-Vega et al. 2025 [47], when adding lignin as a crosslinker, due to their important volumes of available hydroxyl groups, which can form hydrogen bonds with matrices, like PVA, rich in OH groups [48,49].

Regarding the composites, the sample including CNT exhibited stiffer behavior than the CF-PVA-NL sample, consistent with Vidal-Vega et al., 2024 [49]. Furthermore, the sample containing graphene showed the highest rigidity, which makes sense because graphene has been widely studied as a multifunctional composite for its thermal storage capability [50,51].

In the case of tan δ results, the samples ranged from 0.64 (CF-PVA) to 0.86 (CF-PVA-NL, CF-PVA-NL-CNT, and CF-PVA-NL-GP). In contrast, the temperature at which the peak of tan δ was reached varied between 342.3 °C (CF-PVA-NL-GP) and 350.8 °C (CF). This demonstrates that, in materials incorporating PVA, the material’s viscoelasticity increased.

#### 3.2.3. Shear Test of Laminar Joints

The shear test results in normal or dry conditioning, boiling, and a 4-day water-immersion cycle are presented in Table 9.

The strength results of the laminar joints bonded with adhesive bases without epoxy resin under dry test conditions ranged from 5 MPa (P3) to 13 MPa (M2 100%), while for the epoxy resin, a value of 10 N/mm^2^ was obtained. For the boiling test condition, the shear strength was 0 MPa; all the samples failed during the cycle, while for epoxy resin, a value of 9 MPa was obtained. And, for the 4-day water-immersion test condition, the shear strength results ranged from 2 MPa (P4) to 13 MPa (M2 100%), while for the epoxy resin, a value of 8 MPa was obtained.

These results indicated that adhesive bases performed well for indoor use (dry condition) and under humid conditions (4-day water immersion cycle), but not for extreme conditions (boiling cycle), due to the high-water solubility characteristic of PVA [52], which can occur in outdoor applications and under adverse weather conditions, while the control sample ER resisted every condition. Therefore, mixtures of the adhesive bases with a commercial epoxy resin were prepared to enhance their use under extreme conditions. When reviewing these results, it can be observed that, for the extreme conditioning, a significant and satisfactory increase in strength is noted for the 50/50 mixing ratio in all adhesive samples, from M1 to M8, with strength averages ranging from 4 MPa (M8 50%) to 11 MPa (M3 50%). This is consistent with the study by Nurhayati et al. [53] (2025), who reported high ER performance in shear strength, thereby ensuring reliable bonding between CFRP and laminated wood. Nevertheless, some results for the composite samples are higher than those for the ER on its own, and this might be related to the nanoparticles’ hydrophobic properties [24,54].

In general, various samples manufactured with the adhesive bases yielded higher results than the control sample (ER), which was the intended comparison. In addition, some of the composite samples met the class D4 requirement, which was important for evaluating the moisture resistance of the adhesive bases.

#### 3.2.4. Tensile Strength of Carbon Fiber Coated with Adhesive Bases

The results of the tensile strength of carbon fiber coated with adhesive bases are presented in Table 10.

The tensile strength of the coated carbon fiber varied from 492.66 MPa (P1) to 1000.56 MPa (M2). Additionally, it can be observed that this mechanical property increases when nanoparticles of NSiO_2_ and NTiO_2_ are added in most cases, compared to samples P3 and P4. There is also a difference in the results when analyzing the percentage of NSiO_2_ and NTiO_2_; the tensile strength is higher when the samples are composed of 0.01% of NSiO_2_ and NTiO_2_ (M5, M6, M7, and M8) compared with the samples composed of 0.05% of NSiO_2_ and NTiO_2_ (M1, M2, M3, and M4). Another difference is observed: samples composed of GP exhibited higher tensile strength than samples composed of CNT in most cases. These results are consistent with the investigation by Gallego et al. 2013 [55], who compared the tensile performance of graphene and CNT, obtaining better results with graphene, although in their study, they combined the nanoparticles with epoxy resin rather than PVA.

The results obtained in the evaluated properties (characterization of the adhesive bases, dynamic mechanical analysis, shear test of laminar joints, and tensile test of composites made of carbon fiber impregnated with the adhesive bases) indicate that the samples of the adhesive bases of the composites (M1 to M8) showed favorable results for adhesive use, which is favored in 50/50 mass mixtures with a commercial epoxy resin.

Therefore, it has been decided to move on to the next stage with adhesive matrices M5, M6, M7, and M8, since, unlike matrices M1, M2, M3, and M4, they use a smaller amount of nano silicate and nano titanium in their synthesis (from 0.05% to 0.01%), mainly due to the high costs of the nano silicate and nano dioxide titanium and the process to reduce their size to nanoparticles.

In addition to the four adhesive matrices mentioned above, 50/50 epoxy resin mixtures will be included. This will result in a total of eight samples.

### 3.3. Evaluation of Glued-Laminated Radiata Pine Beams at Laboratory Scale

#### 3.3.1. Physical Properties of Glulam Beams Result

The results of the physical properties evaluated in every sample, including density and moisture content, are presented in Table 11.

About the characterization of the glulam beam samples, the density values varied from 437.99 kg/m^3^ (M6) to 468.19 kg/m^3^ (ER). There were no statistically significant differences between the samples with 0% ER (M5, M6, M7, and M8) and those with 50% ER.

The moisture content values varied from 7.91% (M7+ER) to 9.26% (M8), all within the expected range.

#### 3.3.2. Mechanical Properties of Glulam Beams Result

**Figure 8:**
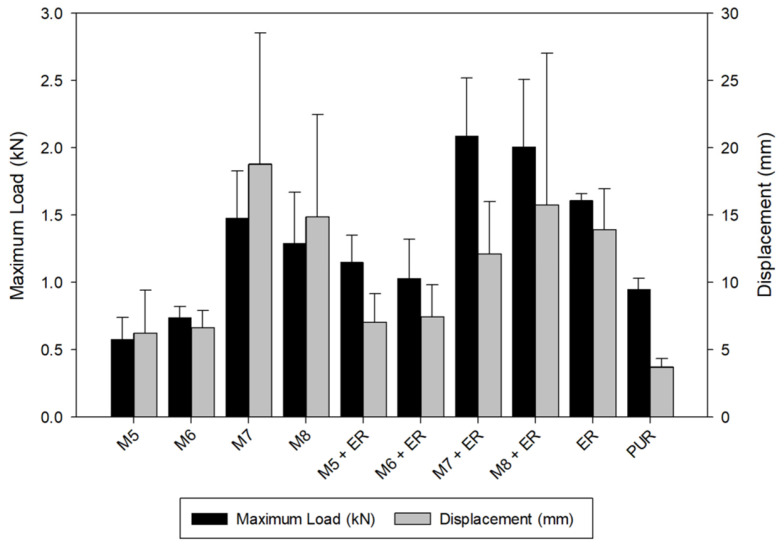
Load–displacement chart of the samples.

The results of the mechanical properties, obtained from the bending performance, evaluated in each sample, are presented in Table 12 and in Figure 8.

Based on the results obtained in the mechanical performance evaluation of glulam beams reinforced with different formulations of high-performance composite, through bending tests, the following analysis can be drawn:

The average values of flexural stiffness (MOE) ranged from 4856 MPa (M5) to 7745 MPa (ER). Statistically significant differences can be observed when comparing the glulam beam samples manufactured with the adhesive bases (M5, M6, and M8) and the glulam beam samples manufactured with the adhesive bases with 50% of epoxy resin; the last ones (M5+ER, M6+ER, and M8+ER) presented higher values and presented a similar performance compared to the glulam beam sample manufactured with ER and PUR adhesives. There is an exception: the sample without ER (M7) showed higher values of flexural stiffness (MOE) and displacement than the sample with epoxy resin (M7+ER).

On the other hand, the average flexural strength (MOR) values ranged from 24.69 MPa (M5) to 50.97 MPa (ER). For this mechanical property, the same behavior was observed as for the MOE property, comparing the results of glulam beams fabricated with adhesive bases with and without 50% ER.

For the maximum load results, the average values ranged from 0.58 kN (M5) to 2.09 kN (M7+ER), and for the displacement results, the average values ranged from 3.73 mm (PUR) to 18.79 mm (M7). A tendency can be observed: samples with adhesive bases containing CNT present lower maximum load and displacement than samples with adhesive bases containing GP. On the other hand, it can also be observed that glulam beam samples manufactured with adhesive bases containing ER in their composition exhibited higher performance than those manufactured with adhesive bases without ER.

Then, the epoxy resin provided the glulam beam samples with a better flexural performance, considering higher values of stiffness, flexural strength, and maximum load capacity. It should be noted that the highest stiffness (MOE) and strength (MOR) in beams reinforced with the different high-performance composite formulations were observed in those whose mixtures contained 50% epoxy resin. Epoxy resin is a structural thermosetting adhesive with high mechanical and thermal properties [56]; nevertheless, there is a strong influence on epoxy resin when it is filled with a nanoparticle, and carbon nanomaterials are widely used as epoxy fillers, since they improve its mechanical properties [57,58]. In this case, the graphene contribution (samples M7 and M8) provided better flexural performance than the CNT contribution (samples M5 and M6). These results are consistent with the investigation by Gallego et al. 2013 [55], who compared the mechanical properties of graphene- and CNT-filled epoxy nanocomposites, obtaining the highest flexural values with graphene.

On the other hand, comparing the PVA and PUR flexural performance, PUR presented higher values, which was demonstrated by the study of Arum et al. 2021 [59], who compared the bending strength performance of PVA and PUR adhesive in glulam beams, concluding that PUR is much more effective due to its binding power and ensures proper bonding of the laminates.

Even though epoxy resin and polyurethane possess stiffer properties than PVA, which provides elasticity to the system, an important quality for a glulam beam, PVA is a nontoxic, biodegradable, and biocompatible polymer [60]; hence, it is a good alternative to replace part of the epoxy resin.

In the next step of this investigation, it could be interesting to try PVA with a higher molecular weight in order to obtain higher elasticity and flexural capacity results [60].

## 4. Conclusions

Through this investigation, a high-performance composite of carbon fiber and nanomaterials was manufactured, improving the mechanical properties of glulam beams.

From the first stage of the investigation, it was concluded that the composites (M1 to M8) showed favorable results for adhesive use, according to thermal and mechanical properties evaluated; therefore, the adhesive bases with a smaller amount of nano silicate and nano titanium were selected, and in addition to the matrices mentioned above, mixtures with 50/50 epoxy resin were included, resulting in a total of eight adhesive bases.

In the second stage, glulam beams were fabricated and subjected to mechanical tests, in which samples reinforced with graphene stood out, while samples mixed with epoxy resin showed flexural stiffness and strength values statistically equivalent to those of the control samples prepared with commercial wood adhesives. It is also important to highlight the performance of the samples M7 (PVA (7.5%) + NL (0.01%) + GP (0.01%) + NSiO_2_ (0.01%)) and M8 (PVA (7.5%) + NL (0.01%) + GP (0.01%) + NTiO_2_ (0.01%)), which are not mixed with epoxy resin and showed statistically the same flexural performance as epoxy resin, in terms of maximum load and displacement.

Finally, it can be concluded that this new high-performance composite is a comparable alternative to hazardous commercial adhesives, achieving lower values that are close to those of the control sample, which is the most commonly used when reinforcing wood products with engineering fibers.

## Figures and Tables

**Table 1:** Identification and abbreviations of the adhesive bases.

ID	Adhesive Bases
ER	Epoxy resin S330
P1	PVA
P2	PVA + NL
P3	PVA _(7.5%)_ + NL _(0.01%)_ + CNT _(0.01%)_
P4	PVA _(7.5%)_ + NL _(0.01%)_ + GP _(0.01%)_
M1	P3 + NSiO_2 (0.05%)_
M2	P3 + NTiO_2 (0.05%)_
M3	P4 + NSiO_2 (0.05%)_
M4	P4 + NTiO_2 (0.05%)_
M5	P3 + NSiO_2 (0.01%)_
M6	P3 + NTiO_2 (0.01%)_
M7	P4 + NSiO_2 (0.01%)_
M8	P4 + NTiO_2 (0.01%)_

**Table 2:** Characterization of adhesive blends.

M1	M2	M3	M4	M5	M6	M7	M8
100%M1 0%ER	100%M2 0%ER	100%M3 0%ER	100%M4 0%ER	100%M5 0%ER	100%M6 0%ER	100%M7 0%ER	100%M8 0%ER
75%M1 25%ER	75%M2 25%ER	75%M3 25%ER	75%M4 25%ER	75%M5 25%ER	75%M6 25%ER	75%M7 25%ER	75%M8 25%ER
50%M1 50%ER	50%M2 50%ER	50%M3 50%ER	50%M4 50%ER	50%M5 50%ER	50%M6 50%ER	50%M7 50%ER	50%M8 50%ER

**Table 3:** Samples evaluated in dynamical thermo-mechanical analysis.

ID	Description
CF	Carbon fiber
CF-PVA	Carbon fiber coated with PVA adhesive
CF-PVA-NL	Carbon fiber coated with PVA and nanolignin
CF-PVA-NL-CNT	Carbon fiber coated with PVA, nanolignin, and carbon nanotubes
CF-PVA-NL-GP	Carbon fiber coated with PVA, nanolignin, and graphene

**Table 4:** Requirements of the shear test of class D4 (UNE-EN 204).

Conditioning Cycle	Shear Strength (MPa)
Dry condition	≥10
Boiling	≥4
4-day water immersion	≥4

**Table 5:** Composite identification for the beams manufacturing.

ID	Description
PUR *	PUR Henkel
ER	ER Sikadur 330+CF
M5	PVA+L+CNT+SiO_2_+CF
M6	PVA+NL+CNT+NTiO_2_+CF
M7	PVA+NL+GP+NSiO_2_+CF
M8	PVA+NL+GP+NTiO_2_+CF
M5+ER	PVA+NL+CNT+NSiO_2_+ER Sikadur330+CF
M6+ER	PVA+NL+CNT+NTiO_2_+ER Sikadur330+CF
M7+ER	PVA+NL+GP+NSiO_2_+CF+ER Sikadur330+CF
M8+ER	PVA+NL+GP+NTiO_2_+CF+ER Sikadur330+CF

*: control sample without composite reinforcement; NL: nanolignin; CNT: carbon nanotubes; GP: graphene; CF: carbon fiber; ER: epoxy resin.

**Table 6:** Characterization of adhesive bases.

ID	Viscosity (cP)	pH	Density (g/mL)	Initial Degradation Temperature (°C)	Mass Loss Initial Thermal Degradation (%)
P1	1140	6.29	1.04	284.84	83.11
P2	1280	6.62	1.05	280.12	83.90
P3	1820	6.33	1.02	288.49	84.47
P4	2740	6.39	1.02	280.44	84.97
M1	2040	9.91	1.06	263.30	49.03
M2	1420	6.53	1.03	285.44	81.13
M3	1020	10.05	1.04	266.06	52.71
M4	1180	6.60	1.02	277.74	81.34
M5	2880	7.04	1.01	276.47	78.67
M6	2520	6.80	1.05	273.77	71.69
M7	1580	7.19	1.01	284.71	84.86
M8	2160	6.34	1.01	272.92	84.11

**Table 7:** Dynamical thermo-mechanical analysis results: storage modulus (E′), tanδ, and temperatures (°C).

ID	Storage Modulus, E′ (GPa)	Temperature, E′ (°C)	Tan δ	Temperature, Tan δ (°C)
CF	333.83	289.5	0.81	350.8
CF-PVA	26.80	298.8	0.64	335.5
CF-PVA-NL	40.35	303.0	0.86	347.2
CF-PVA-NL-CNT	57.28	332.2	0.86	346.7
CF-PVA-NL-GP	113.42	307.1	0.86	342.3

**Table 8:** References of storage modulus of PVA and CF, according to the cited authors.

Sample Description	Reference	E′ (GPa)
PVA modified with microcrystalline cellulose	Vineeth et al. 2023 [39]	5.04
PVA crosslinked with fumaric acid	Agrawal et al. 2023 [40]	5.25
Polyacrylonitrile CF modified with tannins, FeCl_3_, cellulose nanocrystal, epoxy resin, and PVA	Quan et al. 2024 [41]	35.60
CFRP modified with graphene	Kaftelen-Odabaşı. 2023 [42]	~40

**Table 9:** Shear test results under normal or dry conditioning, boiling, and 4-day water-immersion cycles.

ID	Dry Condition				Boiling Cycle				4-Day Water Immersion			
	Shear Strength (MPa)		Wood Failure (%)		Shear Strength (MPa)		Wood Failure (%)		Shear Strength (MPa)		Wood Failure (%)	
	Avg.	St. Dev.	Avg.	St. Dev.	Avg.	St. Dev.	Avg.	St. Dev.	Avg.	St. Dev.	Avg.	St. Dev.
ER	10 ^ghijk^	2	86 ^g^	30	9 ^ef^	3	71 ^d^	27	8 ^ijkl^	2	56 ^gh^	25
P1	5 ^abc^	2	7 ^abc^	7	-	-	-	-	2 ^ab^	2	6 ^ab^	11
P2	9 ^defghij^	3	7 ^ab^	15	-	-	-	-	4 ^cdef^	3	11 ^abc^	32
P3	5 ^ab^	3	13 ^abc^	14	-	-	-	-	4 ^bcdef^	2	13 ^abc^	21
P4	7 ^bcdefg^	3	21 ^abcd^	27	-	-	-	-	2 ^a^	1	8 ^ab^	9
M1100%	8 ^bcdefgh^	3	42 ^de^	45	-	-	-	-	2 ^abc^	2	13 ^abc^	17
M1 75%	9 ^defghij^	7	12 ^abc^	16	-	-	-	-	1 ^abcd^	0	5 ^abcde^	0
M1 50%	7 ^abcdefg^	4	33 ^cde^	25	6 ^cd^	4	42 ^bc^	34	8 ^hijk^	6	44 ^efgh^	27
M2100%	13 ^lmn^	4	29 ^bcd^	23	-	-	-	-	13 ^no^	2	61 ^h^	39
M2 75%	4 ^a^	2	4 ^a^	4	-	-	-	-	8 ^ijkl^	4	0 ^a^	0
M2 50%	8 ^cdefghi^	3	45 ^de^	38	6 ^bc^	2	39 ^abc^	27	9 ^jklm^	4	56 ^fgh^	34
M3100%	11 ^ijklm^	3	41 ^de^	28	-	-	-	-	10 ^klm^	5	62 ^h^	42
M3 75%	11 ^jklm^	4	28 ^abcd^	35	10 ^def^	1	53 ^abcd^	46	9 ^ijklm^	2	33 ^cdef^	29
M3 50%	14 ^mn^	3	57 ^ef^	31	11 ^cd^	2	57 ^cd^	35	10 ^lm^	3	33 ^cdef^	43
M4100%	7 ^bcdefg^	2	17 ^abc^	16	-	-	-	-	5 ^defg^	2	15 ^abcd^	12
M4 75%	15 ^n^	5	44 ^de^	36	8 ^abcd^	4	44 ^abcd^	29	14 ^o^	3	38 ^defg^	29
M4 50%	12 ^klmn^	5	93 ^g^	10	10 ^cd^	2	51 ^cd^	44	11 ^mn^	2	66 ^h^	40
M5100%	9 ^fghij^	3	22 ^abcd^	28	-	-	-	-	9 ^ijkl^	2	28 ^bcde^	24
M5 75%	8 ^defghij^	3	43 ^de^	29	-	-	-	-	5 ^fg^	2	19 ^abcd^	16
M5 50%	6 ^abcde^	3	26 ^abcd^	31	5 ^abc^	2	31 ^abc^	28	7 ^ghij^	1	22 ^abcde^	23
M6100%	10 ^hijkl^	4	41 ^de^	43	-	-	-	-	5 ^efg^	3	11 ^abc^	28
M6 75%	10 ^ghijk^	4	33 ^cde^	34	-	-	-	-	8 ^ijk^	2	14 ^abc^	31
M6 50%	9 ^efghij^	3	29 ^bcd^	30	6 ^bc^	2	14 ^a^	16	7 ^ghi^	1	19 ^abcd^	26
M7100%	5 ^abc^	2	9 ^abc^	16	-	-	-	-	2 ^ab^	1	4 ^a^	5
M7 75%	6 ^abcd^	3	32 ^cd^	34	2 ^a^	1	9 ^a^	11	4 ^bcdef^	3	9 ^abc^	19
M7 50%	8 ^defghij^	2	71 ^fg^	31	7 ^cd^	2	57 ^cd^	27	7 ^ghi^	2	28 ^bcde^	19
M8100%	6 ^abcdef^	3	11 ^abc^	15	-	-	-	-	3 ^abcde^	2	8 ^ab^	13
M8 75%	6 ^abcde^	2	7 ^ab^	5	-	-	-	-	3 ^abcdef^	1	7 ^ab^	8
M8 50%	9 ^fghij^	3	78 ^fg^	29	4 ^ab^	2	21 ^ab^	15	6 ^fgh^	2	22 ^abcde^	25

The superscripts ^a^, ^b^, ^c^, ^d^, ^e^, ^f^, ^g^, ^h^, ^i^, ^j^, ^k^, ^l^, ^m^, ^n^, and ^o^ above the means indicate statistically significant differences between the samples, according to the LSD test at the 95% probability level.

**Table 10:** Results of the tensile strength test of carbon fiber coated with adhesive bases.

ID	Tensile Strength (MPa)	
	Avg.	St. Dev.
P1	492.66 ^a^	66.65
P2	626.58 ^ab^	41.84
P3	523.92 ^ab^	73.30
P4	631.76 ^ab^	125.78
M1	592.08 ^ab^	46.74
M2	1000.56 ^c^	94.31
M3	596.76 ^ab^	112.46
M4	694.38 ^b^	71.55
M5	665.72 ^b^	121.75
M6	693.38 ^b^	113.34
M7	933.42 ^c^	94.84
M8	931.98 ^c^	353.56

The superscripts ^a^, ^b^, and ^c^ above the means indicate statistically significant differences between the samples, according to the LSD test at the 95% confidence level.

**Table 11:** Results of physical properties: density and moisture content of each sample.

ID	Density (kg/m^3^)		Moisture Content (%)	
	Avg.	St. Dev	Avg.	St. Dev
PUR	443.49 ^ab^	11.95	8.00 ^a^	1.10
ER	468.19 ^c^	11.41	8.50 ^abc^	0.55
M5	454.33 ^abc^	21.16	9.00 ^bc^	0.89
M6	437.99 ^a^	9.32	9.00 ^abc^	1.22
M7	455.83 ^abc^	22.78	8.62 ^abc^	0.93
M8	454.05 ^abc^	17.75	9.26 ^c^	0.99
M5+ER	448.78 ^abc^	18.27	8.00 ^ab^	0.89
M6+ER	453.62 ^abc^	13.98	8.17 ^ab^	0.75
M7+ER	461.15 ^bc^	20.04	7.91 ^a^	1.30
M8+ER	458.69 ^abc^	24.24	8.01 ^ab^	0.60

The superscripts ^a^, ^b^, and ^c^ above the means indicate statistically significant differences between the samples, according to the LSD test at the 95% confidence level.

**Table 12:** Results of mechanical properties: flexural stiffness (MOE) and strength (MOR) of each sample.

ID	MOE (MPa)		MOR (MPa)		Maximum Load (kN)		Displacement (mm)	
	Avg.	St. Dev	Avg.	St. Dev	Avg.	St. Dev	Avg.	St. Dev
PUR	7472 ^c^	321.64	45.43 ^ef^	5.01	0.95 ^bc^	0.08	3.73 ^a^	0.63
ER	7745 ^cd^	394.78	50.97 ^f^	4.71	1.61 ^f^	0.05	13.94 ^cd^	3.02
M5	4856 ^a^	483.42	24.69 ^a^	8.73	0.58 ^a^	0.16	6.25 ^a^	3.18
M6	5185 ^a^	337.01	32.63 ^abcd^	1.76	0.74 ^ab^	0.08	6.64 ^ab^	1.26
M7	5974 ^b^	607.36	30.57 ^abc^	7.44	1.48 ^ef^	0.35	18.79 ^d^	9.74
M8	5169 ^a^	510.96	26.43 ^ab^	8.48	1.29 ^def^	0.38	14.87 ^cd^	7.59
M5+ER	7597 ^c^	493.42	45.55 ^ef^	6.02	1.15 ^cde^	0.20	7.05 ^ab^	2.11
M6+ER	7404 ^c^	709.95	38.05 ^cde^	10.07	1.03 ^bcd^	0.29	7.46 ^ab^	2.37
M7+ER	5222 ^d^	647.57	42.89 ^bcde^	7.52	2.09 ^g^	0.43	12.12 ^bc^	3.89
M8+ER	7678 ^cd^	580.54	42.02 ^def^	11.10	2.01 ^g^	0.50	15.76 ^cd^	11.29

The superscripts ^a^, ^b^, ^c^, ^d^, ^e^, ^f^ and ^g^ above the means correspond to statistically significant differences between the samples, according to the LSD test with a 95% probability.

## Data Availability

The data are unavailable due to privacy restrictions associated with an ongoing investigation project.

## Abbreviations

The following abbreviations are used in this manuscript:

CFRP	Carbon fiber-reinforced polymer
CF	Carbon fiber
NL	Nanolignin
CNT	Carbon nanotube
GP	Graphene
PVA	Polyvinyl alcohol
ER	Epoxy resin
PUR	Polyurethane
